# Supplementary material for: Structural basis for membrane recruitment of ATG16L1 by WIPI2 in autophagy
Source: eLife. 2021 Sep 10;10:e70372. doi: 10.7554/eLife.70372 (PMC8455133; doi:10.7554/eLife.70372)
Supplement: Supplementary file 1. [file elife-70372-supp1.docx]

**Supplementary File 1.  Data collection and refinement statistics.**

|  | **WIPI2d** |
| --- | --- |
| **Data Collection Statistics** |  |
| Wavelength | 0.9794Å |
| Resolution range | 38.29  - 1.85 (1.916  - 1.85) |
| Space group | *I* 2 |
| Unit cell | 117.8 49.1 120.1 90 95.9 90 |
| Total reflections | 126271 |
| Unique reflections | 55829 (1711) |
| Multiplicity | 3.3 |
| Completeness (%) | 97.6 (97.8) |
| Mean I/sigma(I) | 6.9 (2.7) |
| Wilson B-factor | 18 |
| R-merge | 0.05 (0.031) |
| R-meas | 0.098 |
| R-pim | 0.068 |
| CC1/2 | 0.996 |
|  |  |
| **Refinement statistics** |  |
| Reflections used in refinement | 55472 (5022) |
| Reflections used for R-free | 885 (85) |
| R-work | 0.1830 (0.2570) |
| R-free | 0.2188 (0.3225) |
| Number of non-hydrogen atoms | 5666 |
| macromolecules | 5041 |
| solvent | 625 |
| Protein residues | 658 |
| RMS(bonds) | 0.007 |
| RMS(angles) | 0.97 |
| Ramachandran favored (%) | 97.99 |
| Ramachandran allowed (%) | 2.01 |
| Ramachandran outliers (%) | 0 |
| Rotamer outliers (%) | 1.83 |
| Clashscore | 4.15 |
| Average B-factor | 25.37 |
| macromolecules | 24.26 |
| solvent | 34.32 |

*Statistics for the highest-resolution shell are shown in parentheses.
