# Supplementary material for: Structural basis for membrane recruitment of ATG16L1 by WIPI2 in autophagy
Source: eLife. 2021 Sep 10;10:e70372. doi: 10.7554/eLife.70372 (PMC8455133; doi:10.7554/eLife.70372)
Supplement: Supplementary file 2. [file elife-70372-supp2.docx]

**Supplementary File 2: Oligos used for cloning**

| Lab Numbering | Sequence | Purpose |
| --- | --- | --- |
| 165 | GCTGTGAAGCTCAACGAGCAGAGGCTGATAG | WIPI2d R108E cloning |
| 166 | CTATCAGCCTCTGCTCGTTGAGCTTCACAGC | WIPI2d R108E cloning |
| 167 | GTACATCCACAACATTGAGGACATGAAGGTGCTGC | WIPI2d R125E cloning |
| 168 | GCAGCACCTTCATGTCCTCAATGTTGTGGATGTAC | WIPI2d R125E cloning |
| 169 | GATTGTTCTCCAGCAGCGAAGTGGCCATCGTGAGC | WIPI2d L69E cloning |
| 170 | GCTCACGATGGCCACTTCGCTGCTGGAGAACAATC | WIPI2d L69E cloning |
| 171 | CAAGGAAGCTAAAGGTTTGCGAATTTAAGAAGGGAACTGAGATC | WIPI2d H85E cloning |
| 172 | GATCTCAGTTCCCTTCTTAAATTCGCAAACCTTTAGCTTCCTTG | WIPI2d H85E cloning |
| 173 | GTTTGCCACTTTAAGGAGGGAACTGAGATC | K88E cloning |
| 174 | GATCTCAGTTCCCTCCTTAAAGTGGCAAAC | K88E cloning |
| 175 | GAAGGGAACTGAGATCGAAAACTACAGCTACTCC | C93E cloning |
| 176 | GGAGTAGCTGTAGTTTTCGATCTCAGTTCCCTTC | C93E cloning |
| 177 | CTTTAAGAAGGGAACTGAGGAATGCAACTACAGCTACTCC | I92E cloning |
| 178 | GGAGTAGCTGTAGTTGCATTCCTCAGTTCCCTTCTTAAAG | I92E cloning |
| 179 | CATTCGGGACATGGAGGTGCTGCATAC | K128E cloning |
| 180 | GTATGCAGCACCTCCATGTCCCGAATG | K128E cloning |
| 98 | CAA ACT CGA GAC TGT GGG ATC GGG ATC GTT CAA CCA GGG CAG AG | loop del (263-295, GS linker) in WIPI2d pCAG cloning |
| 99 | CTC TGC CCT GGT TGA ACG ATC CCG ATC CCA CAG TCT CGA GTT TG | loop del (263-295, GS linker) in WIPI2d pCAG cloning |
| 75 | GAA TTC CTC GAT CGA CGG TAT CGA TGC | WIPI2d pCAG backbone forward cloning |
| 76 | GTT AAT TAA TTA AGA TAT CAC CCG GGT C | WIPI2d pCAG backbone reverse cloning |
